# Ассоциация структуры глюкокортикоидного рецептора и полиморфных вариантов гена <i>NR3C1</i> с метаболическими нарушениями

**DOI:** 10.14341/probl13160

**Published:** 2023-02-25

**Authors:** С. С. Бровкина, И. С. Джериева, Н. И. Волкова, Т. П. Шкурат, З. А. Гончарова, Е. В. Машкина, И. Б. Решетников

**Affiliations:** Ростовский государственный медицинский университет; Ростовский государственный медицинский университет; Ростовский государственный медицинский университет; Южный федеральный университет; Ростовский государственный медицинский университет; Южный федеральный университет; Ростовский государственный медицинский университет

**Keywords:** глюкокортикоидный рецептор, полиморфные варианты гена NR3C1, метаболические нарушения, сердечно-сосудистые заболевания

## Abstract

Глюкокортикоидная терапия широко используется при лечении различных патологий. Чувствительность к глюкокортикоидам (ГК) оказывает серьезное влияние не только на эффективность их действия, но и на выраженность побочных эффектов, формирование факторов риска и развитие сердечно-сосудистых  заболеваний (ССЗ). Вариабельность чувствительности к ГК обуславливает различные фенотипы и выраженность  метаболических нарушений, лежащих в основе ССЗ. Среди них можно выделить снижение мышечной массы и силы, ожирение, нарушение углеводного, липидного обмена и другие. ГК осуществляют свои эффекты путем связывания с глюкокортикоидным рецептором (ГР), в связи с чем это считается критической  точкой в их действии. В настоящем обзоре представлены данные о значении структуры ГР, рассмотрены основные полиморфные варианты гена ГР NR3C1, ассоциированные с гиперчувствительностью или относительной резистентностью  к ГК в контексте метаболических нарушений и развития ССЗ. Подробно описаны ассоциации 4 наиболее изученных полиморфных локусов гена ГР NR3C1 с метаболическими рисками: BclI (rs41423247), N363S (rs56149945), ER22/23EK (rs6189/rs6190), GR-9ß (rs6198). Их определение может способствовать  уточнению прогноза  как эффективности ГК, так и развития метаболических нарушений, и последующей ранней коррекции факторов риска ССЗ.

## Abstract (en)

Glucocorticoid therapy is widely used in the treatment of various pathologies. Sensitivity to glucocorticoids  (GC) has a serious impact not only on the effectiveness of their action, but also on the severity of side effects, the formation of risk factors and the development  of cardiovascular diseases (CVD). Variability of sensitivity to GC causes different phenotypes and severity of metabolic disorders underlying  CVD. Among  them, one can distinguish  a decrease in muscle mass and strength, obesity, glucose and lipid metabolism impairment, and others. Glucocorticoids carry out their effects by binding to the glucocorticoid receptor (GR), and therefore this is considered a critical point in their action. This review presents data on the significance of the glucocorticoid  receptor structure, examines the main single nucleotide polymorphisms (SNP) of the NR3C1 gene associated with hypersensitivity  or relative resistance to glucocorticoids  in the context of metabolic disorders and the development of CVD. The association of the four most studied SNP of the GR gene with metabolic risks is described in detail: BclI (rs41423247), N363S (rs56149945), ER22/23EK (rs6189/rs6190), GR-9ß (rs6198). Their determination can contribute to clarifying the prognosis of both the effectiveness of GC and the development of metabolic disorders, and subsequent early correction of CVD risk factors.

Сердечно-сосудистые заболевания (ССЗ) вызывают более трети всех смертей в мире по данным ВОЗ [1]. В настоящее время существует возможность снизить частоту их развития при воздействии на факторы риска: гиподинамию, курение, употребление большого количества соли, злоупотребление алкоголем, нездоровое питание, высокое артериальное давление (АД), нарушение углеводного, липидного обмена, избыточную массу тела и ожирение [2]. Ответственность за приверженность здоровому образу жизни и воздействие на поведенческие факторы риска в основном возлагается на самого пациента. Однако на современном этапе особое значение приобретают как ятрогенный фактор риска, зависящий от пациента и врача, так и генетическая предрасположенность, на которую пока мы воздействовать не можем.

Применение глюкокортикоидов (ГК) серьезно влияет на метаболические процессы и приводит к побочным эффектам, которые являются факторами риска ССЗ [3–5]. Международное медицинское сообщество было особенно озабочено необходимостью и целесообразностью применения ГК в свете пандемии вируса SARS-CoV-2 [6]. Отмечено, что даже однократное введение ГК может вызывать негативные последствия, в том числе острую стероидную миопатию [7]. Это осложнение получало немного внимания, но в настоящее время ему придается все большее значение. Проведены исследования, рассматривающие состояние мышечной ткани как самостоятельный фактор риска и предиктор негативного исхода различных заболеваний, в том числе хронической сердечной недостаточности, хронической болезни почек и других патологий [8][9].

Следовательно, последствия применения ГК, в том числе стероидная миопатия, увеличивают риск развития ССЗ, ухудшают качество жизни, а также могут привести к фатальным исходам. Побочные эффекты ГК также наносят существенный экономический урон. Итальянские исследователи проанализировали с помощью динамической имитационной модели с использованием данных Итальянской национальной системы здравоохранения (INHS) стоимость лечения побочных эффектов оральных ГК. Учитывалось возникновение диабета 2 типа, ожирения, дислипидемии, бессонницы и других состояний. При этом стоимость лечения в течение года для одного пациента начиналась от 1065€, а при применении оральных ГК на фоне бронхиальной астмы средней и тяжелой степеней тяжести она возрастала на 892€ и 606€ соответственно [10].

При этом благодаря своей эффективности, разнообразию форм и удобству применения ГК входят в число одних из самых часто назначаемых препаратов. Так, во Франции распространенность перорального применения ГК колебалась от 14,7 до 17,1% населения [11]. Однако частота назначения и применения ГК в России до настоящего времени остается неотслеженной [12].

Среди факторов риска ССЗ есть те, на которые мы не можем повлиять, — например, генетическая предрасположенность и наследственный анамнез. В свете распространенности применения ГК приобретает особенное значение гиперчувствительность как к эндогенным, так и к экзогенным ГК, обусловленная полиморфными вариантами гена глюкокортикоидного рецептора (ГР). Это приводит к более выраженным проявлениям нежелательных побочных эффектов и метаболическим нарушениям. Существуют и полиморфные локусы, ассоциированные с устойчивостью к ГК, что проявляется неспособностью ГК оказывать желаемое воздействие на ткани-мишени, ограничивая эффективность лечения. Вариабельность гена ГР может сказываться, в том числе, на функционировании сердечно-сосудистой системы [13][14].

Сочетание поведенческих факторов риска с генетическими изменениями на фоне терапии ГК повышает риски ССЗ. В связи с чем для повышения безопасности и эффективности лечения с применением ГК, разработки новых подходов к ведению пациентов, получающих данные препараты, необходимо иметь всестороннее представление о факторах, влияющих на метаболизм и эффекты ГК. Поскольку связывание с ГР является критическим процессом в действии ГК, этот обзор направлен на то, чтобы представить обновленную информацию о значении вариабельности структуры гена ГР и ее влиянии на сердечно-сосудистую систему и метаболические процессы, обуславливающие риск ССЗ.

При поиске источников были использованы полнотекстовые и реферативно-библиографические базы данных: Embase, Google Scholar, Ensembl.org, Сyberleninka.ru, Library Wiley.com, Web of Science, PubMed, Elsevier (ScienceDirect). Анализ литературы проведен за 30 лет (с 1991 по 2021 гг.). Поиск источников осуществлялся по следующим ключевым словам с соответствующим переводом для англоязычных баз с использованием логических операторов AND, OR: полиморфизмы гена глюкокортикоидного рецептора NR3C1, ожирение, метаболический синдром, дислипидемия, артериальная гипертензия, миопатия, мышечная сила, масса, гипергликемия, инсулинорезистентность, сердечно-сосудистые заболевания. В поиск были включены клинические и оригинальные исследования, систематические обзоры, метаанализы.

## ГЛЮКОКОРТИКОИДНЫЙ РЕЦЕПТОР: ИЗОФОРМЫ

ГР экспрессируется во всех клетках организма с ядрами и кодируется геном ГР NR3C1, который расположен на пятой хромосоме (5q31–32) у человека. ГР действует как лиганд-индуцируемый фактор транскрипции и регулятор других факторов транскрипции, относится к суперсемейству ядерных рецепторов [15].

У человека ген ГР состоит из девяти экзонов. Рецептор имеет несколько изоформ, например, ГРα и ГРβ, которые являются результатом альтернативного сплайсинга экзона 9 [16]. С транскрипта, то есть молекулы мРНК, ГРα может осуществляться инициация трансляции с нескольких старт-кодонов, расположенных в экзоне 2 гена ГР NR3C1, генерируя восемь дополнительных изоформ ГР (ГРα-A, ГРα-B, ГРα-C1, ГРα-C2, ГРα-C3, ГРα-D1, ГРα-D2 и ГРα-D3) [16]. Они демонстрируют некоторые различия, например, изоформа ГР-C повышает восприимчивость к апоптозу в культуре клеток, а ГР-D приводит к относительной устойчивости к апоптозу в тех же условиях [17].

С клинической точки зрения важно понимать, что вариант ГРβ не связывает ГК и действует как естественный доминантный ингибитор изоформы ГРα на многих генах-мишенях, чувствительных к ГК [18]. Способность ГРβ ингибировать активность изоформы ГРα предполагает, что высокие уровни ГРβ могут привести к резистентности к ГК [19]. Воспалительный процесс может увеличить экспрессию β-изоформы. Было показано, что провоспалительные цитокины особенно сильно активируют механизмы альтернативного сплайсинга [20]. Данный факт может частично объяснять случаи недостаточного ответа на ГК-терапию воспалительных заболеваний.

Однако действия изоформы ГРβ не ограничены ее эффектами на ГРα. Выявлено, что ГРβ, кроме ядра, находится в цитоплазме клетки, обладает внутренней активностью и регулирует множество генов, связанных с воспалительным процессом, клеточной коммуникацией, миграцией, каждый из которых независим от ГРα. Кроме того, ГРβ был связан с увеличением миграции клеток и снижением чувствительности к ГК-индуцированному апоптозу [21][22].

ГРβ контролирует рост клеток, метаболические процессы, такие как глюконеогенез и обмен липидов. Несколько сообщений показало, что в клеточных линиях человека и мыши (миобласты, эмбриональные фибробласты, клеточная линия макрофагов и др.) инсулин повышает уровень мРНК и белка ГРβ [23]. Это было подтверждено исследованиями in vivo, в которых отмечена повышенная экспрессия ГРβ в печени генетически диабетических крыс Гото-Какидзаки, характеризующихся значимо повышенным уровнем инсулина по сравнению с контрольной группой [24]. Таким образом, эти исследования показали, что гиперинсулинемия сопровождается повышением количества ГРβ, что может приводить к худшему ответу на ГК-терапию.

Количество ГРβ повышается у мышей в жировой ткани и печени при ожирении, вызванном диетой, насыщенной жирами. Участие ГРβ в функциях печени было дополнительно продемонстрировано у мышей путем сверхэкспрессии ГРβ в печени при стандартном количестве жиров в рационе [25]. В результате происходило накопление триглицеридов в печени и их увеличение в сыворотке крови, тем самым способствуя патогенезу неалкогольной жировой болезни печени (НАЖБП). Также отмечалось увеличение белка синтазы жирных кислот в печени, говорящее об активности образования жирных кислот de novo. Наблюдалось снижение накопления гликогена в печени и повышение гликемии натощак, вероятно, за счет печеночной инсулинорезистентности. Внимание привлекает также снижение активности сигнальных путей, опосредованных печеночными PPARα и фактором роста фибробластов 21, вызывающих при действии ГК липолиз во время голодания. Таким образом, большая экспрессия ГРβ в печени может обуславливать развитие НАЖБП, гипертриглицеридемии, снижение липолиза и адаптации к голоданию.

С учетом описанных механизмов, можно предположить, что ожирение, считающееся обычно следствием избыточного действия и гиперчувствительности к ГК, за счет гиперинсулинемии, увеличения экспрессии ГРβ может самостоятельно приводить к снижению терапевтического эффекта ГК и ухудшению липидного профиля, замыкая порочный круг.

Дополнительные изоформы ГР были связаны с нечувствительностью к ГК. ГРγ проявляет около 50% активности ГРα, ГР-P и ГР-A имеют серьезные нарушения структуры и не связывают ГК, способствуя резистентности к последним. ГР-P при этом модулирует транскрипционную активность ГРα специфичным для клеток образом [23–25].

Таким образом, различные ткани демонстрируют специфичную распространенность изоформ ГР, которая может меняться в ответ на уникальные стимулы, такие как гиперинсулинемия, ожирение, старение и другие. Эти различия имеют, по-видимому, решающее значение для определения тканеспецифического действия ГК. Однако даже изменения одного нуклеотида в последовательности ДНК гена NR3C1 могут сказываться на эффектах ГК. Ассоциация однонуклеотидных полиморфизмов (ОНП), возникающих в результате точечных мутаций в гене ГР, чувствительности к ГК и развития метаболических нарушений, будет подробно рассмотрена ниже.

## ПОЛИМОРФНЫЕ ВАРИАНТЫ ГЕНА ГЛЮКОКОРТИКОИДНОГО РЕЦЕПТОРА

Сообщалось о различных полиморфных локусах гена ГР, связанных с измененной чувствительностью к ГК. Они не были сконцентрированы в каких-то определенных областях гена. Кроме того, некоторые из них обнаруживаются в области гена, не кодирующей белок [26]. Ассоциированными с ССЗ или с лежащими в их основе факторами риска считаются варианты ER22/23EK (rs6189/rs6190), ГР-9β (rs6198), N363S (rs56149945) и BclI (rs41423247). Было показано, что гиперчувствительность к ГК возникала при наличии N363S (rs56149945) и BclI (rs41423247). Полиморфные варианты гена ER22/23EK (rs6189/rs6190), ГР-9β (rs6198) были ассоциированы с резистентностью к ГК в контексте более здорового метаболического профиля [27]. Обобщенные данные приведены в таблице 1.

**Table:** Таблица 1. Ассоциация полиморфизмов гена NR3C1 с метаболическими изменениями

Полиморфизм гена NR3C1	Чувствительность к ГК	Ассоциация	Ссылки
BclI (rs41423247)	Повышенная	Ожирение, дислипидемия, гиперинсулинемия, большая жировая масса, гипергликемия	J. Weaver et al. [29], R. Rosmond et al. [30], R. Giordano et al. [31], A. Tremblay et al. [32], C. Geelen et al. [33], P. Barat et al. [34], C. Kaymak et al. [35], A. Zawiejska et al. [36]
N363S (rs56149945)	Повышенная	Ожирение, гиперинсулинемия, большее отношение ОТ к ОБ, больший ИМТ, метаболический синдром	N. Huizenga et al. [38], A. Di Blasio et al. [39], M. Dobson et al. [41], A. Marti et al. [42], M. Savas et al. [43], E. Wester et al. [44]
ER22/23EK (rs6189/rs6190)	Сниженная	Меньшие концентрации инсулина, ЛПНП, С-реактивного белка, гликемия, прибавка в весе во время беременности; большие безжировая масса тела, мышечная сила, продолжительность жизни	E. van Rossum et al. [46], E. van Rossum et al. [47], E. van Rossum et al. [48], M. Finken et al. [50], R. Bertalan et al. [51]
ГР-9β (rs6198)	Сниженная	Сниженный риск ожирения, тревожность, потребление сахара, большие ЛПВП, мышечная сила	M. Savas et al. [43], B. Liu et al. [53], L. Müller et al. [54], D. Rodrigues et al. [57]

## ПОЛИМОРФНЫЕ ВАРИАНТЫ ГЕНA NR3C1, АССОЦИИРОВАННЫЕ С ГИПЕРЧУВСТВИТЕЛЬНОСТЬЮ К ГЛЮКОКОРТИКОИДАМ

Полиморфный вариант гена NR3C1 ГР BclI (rs41423247) является результатом замены нуклеотидов во 2 интроне (цитозин-гуаниновая трансверсия). Согласно базе данных Ensembl, в европейской популяции идентифицируется со следующими частотами: аллель G — 62%, минорный аллель С — 38%; генотипы GG, GC, CC — 37,4, 49,3, 13,3% соответственно [28]. Наличие мутантного аллеля BclI (rs41423247) ГР ассоциировано с относительной гиперчувствительностью к ГК.

В одной из ранних работ J. Weaver и соавт. [29] показали ассоциацию между наличием BclI (rs41423247) и гомеостазом глюкозы. В исследование были включены 56 женщин с ожирением (средний индекс массы тела (ИМТ) 42 (34–59) кг/м2) и 43 здоровые женщины. Среди гомозигот по мутантному аллелю с ожирением наблюдались значительное повышение концентрации инсулина натощак и повышенный индекс инсулинорезистентности (HOMA) по сравнению с группой, гомозиготной по нормальному аллелю, и гетерозиготами. Авторами была выдвинута одна из первых гипотез о том, что наличие изменений в гене NR3C1 ГР может непосредственно влиять на экспрессию генов ГК-рецепторов или быть связано с модуляцией транскрипционной активности генов, кодирующих белки, ответственные за поддержание гомеостаза глюкозы.

Другие исследования, проведенные с 2000 г., выявили связь между описанным вариантом и метаболическими нарушениями, встречающимися при применении ГК [30][31]. На основании полученных данных можно утверждать, что не только гомозиготы по мутантному аллелю, но и гетерозиготы имели метаболические изменения, эффект являлся аллель-зависимым. В ходе 12-летнего наблюдения в исследовании, проведенном А. Tremblay и соавт. [32], продемонстрировано, что гетерозиготы по полиморфизму BclI (rs41423247) имеют повышенную склонность к накоплению жировой ткани по сравнению с гомозиготами по обоим аллелям. Данные двух голландских когортных исследований (Hoorn и CODAM) С. Geelen и соавт. [33] показали, что гомозиготы по мутантному аллелю имеют значительно больший процент жировой массы тела, ИМТ и более выраженную инсулинорезистентность. К аналогичному выводу привели результаты обследования детей, страдающих ожирением. Двухэнергетическая рентгеновская абсорбциометрия, позволяющая проводить более точные исследования, чем антропометрия, показала, что гомозиготы по мутантному аллелю имели повышенную жировую массу тела [34]. Среди педиатрических пациентов с острым лимфобластным лейкозом также выявлено, что у носителей полиморфного локуса BclI (rs41423247) с большей вероятностью разовьются признаки синдрома гиперкортицизма (например, ожирение, гипертония, диабет) и депрессия во время системной терапии ГК [35]. Приведенные факты могут подтвердить гипотезу о том, что полиморфный вариант BclI (rs41423247) ассоциирован с негативными метаболическими последствиями.

В недавнем исследовании показано, что беременные женщины, гомозиготы по полиморфному варианту BclI (rs41423247), имели больший риск развития гипергликемии [36]. Возможным объяснением данного факта может стать то, что сама беременность является состоянием, характеризующимся инсулинорезистентностью как механизмом адаптации. Наличие полиморфного варианта BclI (rs41423247), вероятно, приводило к чрезмерному усилению изменений, что ассоциировалось с гипергликемией. Клиническое значение определения данного ОНП гена ГР может состоять в выявлении подгрупп пациентов из группы риска метаболических нарушений, которые в наибольшей степени выиграют от разработки персонализированного подхода с более частым лабораторным контролем, рекомендациями по физической нагрузке.

С проявлениями относительной гиперчувствительности к ГК также связывают один из наиболее часто описываемых полиморфных вариантов гена ГР NR3C1 — N363S (rs56149945). Он приводит к транзиционной мутации во 2 экзоне гена NR3C1 1220 нуклеотида (замена аденина на гуанин) и как следствие — к замене в кодоне 363 аспарагина на серин в полипептидной цепи рецептора ГР. Частота аллельных вариантов по локусу N363S (rs56149945) в европейской популяции оценивается как аллель T 98,2%, аллель С 1,8%; генотипы TT — 96,4%, CT — 3,6% [37].

Ассоциация между полиморфным вариантом N363S (rs56149945) гена NR3C1 и избыточной массой тела была показана еще N. Huizenga и соавт. [38]. Гетерозиготные носители имели более высокий ИМТ. Авторы показали, что полиморфный вариант N363S (rs56149945) ассоциирован с повышенной чувствительностью ГР in vivo. Уровни глюкозы натощак перед введением дексаметазона были одинаковыми в группе гетерозигот N363S (rs56149945) и в группе, не имеющей данной мутации, но после введения 1 мг дексаметазона уровень инсулина в плазме значительно увеличился среди пациентов гетерозигот. Предполагалось, что низкие дозы стероидов вызывают рост секреции инсулина для поддержания нормального уровня гликемии. Это дополнительно указывает на то, что носители полиморфного варианта N363S (rs56149945) более чувствительны к ГК.

Еще одно исследование, проведенное командой А. Di Blasio’s [39], подтвердило ассоциацию между ожирением и полиморфным локусом N363S (rs56149945). Были обследованы пациенты с ожирением (средний ИМТ 45,9±0,9 кг/м2) и пациенты с нормальной массой тела. Гетерозиготный вариант N363S (rs56149945) встречался чаще среди пациентов с ожирением. Гетерозиготные по данной мутации женщины с ожирением имели значимо больший ИМТ, чем гомозиготы по нормальному аллелю. Среди мужчин значимых различий выявлено не было. Обладатели обоих полиморфных вариантов BclI (rs41423247) и N363S (rs56149945) имели большие АД систолическое и диастолическое, а также хуже липидный профиль. На основе своих наблюдений А. Di Blasio и соавт. пытались объяснить патофизиологическую роль N363S (rs56149945) в развитии ожирения, предполагая, что повышенная чувствительность носителей этого генетического варианта к ГК может приводить к повышенной секреции инсулина в ответ на гормональный стимул, которая теоретически может лежать в основе усиленного липогенеза и возможного последующего увеличения массы тела.

Связь между полиморфным вариантом N363S (rs56149945) и увеличенной массой тела была поставлена под сомнение R. Rosmond и соавт. [40] и M. Dobson и соавт. [41]. Ученые не выявили взаимосвязи между наличием мутантного аллеля и увеличением массы тела. Однако M. Dobson и соавт. подтвердили более высокие значения отношения окружности талии (ОТ) к окружности бедер (ОБ) среди мужчин гетерозигот.

В 2006 г. A. Marti и соавт. [42] опубликовали метаанализ влияния полиморфного локуса N363S (rs56149945) на ожирение. В него были включены исследования, проведенные среди населения Испании и Германии, предпринята попытка обобщить данные из ранее опубликованных 12 исследований. Общее количество испытуемых составило 5909 человек из разных стран мира. В связи с большой гетерогенностью популяции по массе тела выделено 4 исследования, куда вошли пациенты с ИМТ менее 27 кг/м2. Анализ этой подгруппы показал, что носители мутантного аллеля в выделенной подгруппе имели статистически значимо больший ИМТ при сравнении с гомозиготами по нормальному аллелю. Однако достоверных данных, подтверждающих ассоциацию N363S (rs56149945) с риском ожирения и величиной ИМТ, в итоге получено тоже не было.

Тем не менее в последние годы проведены крупные популяционные исследования, подтверждающие взаимосвязь полиморфных вариантов гена ГР с метаболическими изменениями. В работу группы голландских врачей M. Savas и соавт. [43] включены более 10 тысяч человек. Большая часть пациентов получали ГК топически (ингаляционно, интраназально, накожно), однако были включены и пациенты с системной ГК-терапией. Вероятность развития метаболического синдрома была выше в группе ГК с неизмененным генотипом ГР в 1,64 раза (95% доверительный интервал (ДИ) 1,06–2,55; p=0,027) и 1,43 раза (95% ДИ 1,08–1,91; p=0,013) в группе с гиперчувствительными вариантами гена ГР (BclI (rs41423247), N363S (rs56149945)) по сравнению с пациентами, не получающими ГК. При этом значимой разницы у пациентов с системной ГК-терапией не было выявлено. Такую находку, вероятно, можно объяснить ограниченной выборкой пациентов с системной терапией. ОТ, в свою очередь, была увеличена во всех группах, получающих ГК, вне зависимости от наличия измененных вариантов гена ГР. Была отмечена тенденция к увеличению ОТ в группе с гиперчувствительными генотипами ГР (резистентный генотип ГР (ER22/23EK (rs6189/rs6190), A3669G (rs6198) +2,34 см [ 95% ДИ 0,10–4,59], p=0,040; гомозиготы по нормальному аллелю +3,80 см [ 95% ДИ 1,62–5,97], p<0,001; гиперчувствительный генотип ГР (BclI (rs41423247), N363S (rs56149945) +4,72 см [ 95% ДИ 3,34–6,11], p<0,001)).

V. Wester и соавт. [44] в популяционном исследовании с участием более 12 тысяч человек также показали, что N363S (rs56149945)ассоциирован с увеличением частоты метаболического синдрома, но только у более молодых мужчин (средний возраст 47 лет или ниже; ОШ 1,77; p=0,005) и у людей с низким уровнем образования (ОШ 1,48; P=0,039). Таким образом, выявлены данные, свидетельствующие о связи полиморфных локусов гена ГР NR3C1 с относительной гиперчувствительностью к ГК и метаболическими нарушениями, но существуют и факты, противоречащие этому. В связи с чем большее количество исследований поможет оценить фенотипы данных полиморфизмов и уточнить их роль в формировании кардиометаболических рисков.

## ПОЛИМОРФНЫЕ ВАРИАНТЫ ГЕНА NR3C1, АССОЦИИРОВАННЫЕ С РЕЗИСТЕНТНОСТЬЮ К ГЛЮКОКОРТИКОИДАМ

Полиморфный вариант гена ГР ER22/23EK (rs6189/rs6190) ассоциирован с относительной резистентностью к ГК. Данные изменения вызваны двумя связанными точечными мутациями в соседних нуклеотидах. Первая нуклеотидная замена в 198 положении является синонимичной и не изменяет кодируемую 22 кодоном аминокислоту (глутаминовая кислота). Другая мутация приводит к замене аргинина на лизин в 23 кодоне полипептидной цепи рецептора. В европейской популяции частота аллелей составляет: С — 97%, Т — 3%, а генотипов: СС — 94%, СТ — 6 [45].

Исследования in vivo показали, что носители полиморфизма ER22/23EK (rs6189/rs6190), в отличие от широко описанного N363S (rs56149945), имеют благоприятный метаболический профиль, вероятно, из-за их сниженной чувствительности к влиянию ГК. В исследовании Е. van Rossum и соавт. [46], проведенном с участием 202 пожилых людей, было идентифицировано 18 гетерозигот (8,9%). Дважды проводился супрессивный тест с дексаметазоном, дозы составляли 0,25 мг и 1 мг. Цель исследования состояла в том, чтобы сравнить концентрации кортизола и инсулина в плазме у носителей описанного полиморфного варианта и людей без мутантного аллеля. После введения 1 мг дексаметазона в группе носителей ER22/23EK (rs6189/rs6190) наблюдались повышенные уровни кортизола в плазме. Более того, у носителей данного полиморфизма были ниже концентрации инсулина и липопротеинов низкой плотности (ЛПНП) во время голодания. Наличие мутации было ассоциировано со снижением концентрации инсулина и глюкозы крови до и после супрессивного теста с 1 мг дексаметазона. Таким образом, предположено, что носители мутантного аллеля ER22/23EK (rs6189/rs6190) более устойчивы к влиянию ГК и чувствительны к инсулину, что отражается в виде более благоприятного метаболического профиля.

Е. Van Rossum и соавт. [47] также предприняли попытку проанализировать действие ГК на перераспределение жировой ткани, а также мышечную силу с учетом наличия ER22/23EK (rs6189/rs6190) среди молодых людей в возрасте 13–36 лет. Этот эффект гормонов коры надпочечников играет важную роль в липидном обмене и напрямую связан с чувствительностью к инсулину, развитием потенциальных нарушений гомеостаза глюкозы и, как следствие, сильно влияет на общий сердечно-сосудистый риск. Авторы в течение 23 лет наблюдали за участниками исследования, чтобы сравнить антропометрические параметры носителей описанного полиморфного варианта гена ГР с людьми без мутантного аллеля. Процент носителей ER22/23EK (rs6189/rs6190) был аналогичен таковому из предыдущего исследования и достигал 8%. Было показано, что носители были выше ростом, имели большую безжировую массу тела, большую ОБ и большую мышечную силу. Среди женщин наблюдался более низкий показатель отношения ОТ к ОБ. Разницы в ИМТ или жировой массе при этом замечено в обеих группах не было.

Наблюдения датских ученых показывают, что полиморфный вариант ER23/22EK (rs6189/rs6190) может быть ассоциирован с продолжительностью жизни [48]. Исследование, включившее группу мужчин в возрасте 77,8±3,6 года, показало, что среди лиц без полиморфного варианта ER23/22EK (rs6189/rs6190) за 4-летний период наблюдения процент смертей составил 19,2%, в то время как в группе носителей мутации период наблюдения пережили все. У пациентов с полиморфным вариантом ER23/22EK (rs6189/rs6190) наблюдались более низкие уровни С-реактивного белка, общего холестерина и ЛПНП, что обуславливало лучший сердечно-сосудистый профиль. Важно подчеркнуть у них более высокий процент безжировой массы тела, который связан с большей чувствительностью к инсулину и является важным фактором снижения общего сердечно-сосудистого риска. Описанные отличия в совокупности, вероятно, могли повлиять на большую продолжительность жизни.

В 2006 г. М. Kuningas и соавт. [49] опубликовали несколько противоречивые данные — результаты исследования влияния полиморфных вариантов гена ГР на общий сердечно-сосудистый риск в группе людей старше 85 лет. Носители мутаций ER23/22EK (rs6189/rs6190) имели более высокие уровни HbA1с. Кроме того, у этих пациентов был выявлен повышенный уровень С-реактивного белка. Тем не менее ассоциации с сердечно-сосудистым риском или смертностью ни в одной из групп не было обнаружено.

Иные данные были получены среди детской популяции. Голландские авторы [50] на основе 19-летнего наблюдения за группой недоношенных детей, рожденных до 32-й недели, показали, что наличие полиморфного аллеля связано с более низкой концентрацией инсулина натощак и более низкими значениями индикаторов инсулинорезистентности. Также было выявлено, что у носителей мутаций ER23/22EK (rs6189/rs6190) наблюдался дефицит роста в возрасте до одного года, однако их конечный рост был аналогичен среднему росту населения. В это время в группе недоношенных детей без ER23/22EK (rs6189/rs6190) конечный рост был в среднем на половину стандартного отклонения меньше. Как равенство роста, так и более низкие уровни инсулина, по-видимому, подтверждают гипотезу об относительной резистентности носителей ER23/22EK (rs6189/rs6190) к эффектам ГК и большей чувствительности к инсулину.

Защитная роль полиморфного локуса ER22/23EK (rs6189/rs6190) также наблюдалась группой венгерских ученых R. Bertalan и соавт. [51]. Среди женщин, гетерозиготных носителей данного полиморфного варианта, отмечена меньшая прибавка в весе во время неосложненной беременности. Тем не менее остается открытым вопрос о влиянии пола на фенотипические проявления различных вариантов гена ГР. Зачастую это связано с недостаточным объемом выборки и отсутствием разделения носителей ОНП по полу. Для оценки вклада половой принадлежности необходимо больше исследований с более строгим дизайном.

Полиморфный вариант гена NR3C1 ГР 9β (A3669G или rs6198) также ассоциирован с относительной резистентностью к ГК. Он связан с заменой A на G в 3’-UTR конце экзона 9. Для европейской популяции наблюдается следующая частота аллелей: T — 82,6%, C — 17,4%. Генотипическая структура представляет собой соотношение: TT — 69,2%, CT — 26,8%, CC — 4% [52].

Наличие полиморфного аллеля также ассоциировано с более благоприятным метаболическим профилем и связано со снижением риска ожирения у женщин, снижением уровня общего холестерина и повышением уровня холестерина ЛПВП [53]. В ранее упомянутом исследовании M. Savas et al. [43] отмечают отсутствие или минимально выраженные побочные эффекты применения ГК в группе носителей резистентных полиморфных вариантов, в том числе A3669G (rs6198).

Известно, что мышечная ткань участвует в утилизации глюкозы и играет роль в развитии метаболических нарушений. ГК могут негативно влиять на мышцы, вызывая стероидную миопатию. Коллективом немецких ученых в недавнем исследовании у пациентов с эндогенным синдромом гиперкортицизма была оценена связь полиморфных вариантов гена ГР с состоянием мышечной ткани [54]. Среди пациентов с активным заболеванием носители полиморфного аллеля A3669G (rs6198) имели большую мышечную силу по сравнению с гомозиготами по нормальному аллелю (p=0,006 для доминантной и p=0,021 для недоминантной руки соответственно). В связи с чем можно предположить, что обладатели неизмененного генотипа AA3669 более восприимчивы к избытку ГК, чем носители мутантного аллеля, которые защищены относительной резистентностью к ГК от развития миопатии. Среди мужчин — гомозигот по референсному аллелю ER22/23EK (rs6189/rs6190) была отмечена меньшая мышечная сила относительно пациентов с полиморфным аллелем (p=0,049 для доминантной руки; p=0,027 для недоминантной руки). Полученные данные могут дать объяснение различиям между пациентами при развитии ГК-индуцированной миопатии. Что получает особое значение, учитывая факт корреляции низкой мышечной силы с более высокой смертностью и низким качеством жизни [55].

Мышечная масса, дислипидемия, атеросклероз и другие метаболические процессы в совокупности влияют на гемодинамику. Наличие полиморфного варианта A3669G (rs6198) и изменение артериального давления были отмечены в недавнем исследовании у пациентов с синдромом или болезнью Кушинга, находящихся в длительной ремиссии [56]. Так, у носителей данного варианта гена ГР отмечали более высокий уровень систолического АД и более низкие уровни резистина. Однако полученные данные следует интерпретировать с осторожностью в связи с небольшой выборкой и фактом того, что после лечения часть пациентов получали препараты ГК по поводу развившегося гипокортицизма.

Одним из аспектов, играющих роль в метаболических нарушениях, также являются пищевые привычки и ментальный статус. Было показано, что у пациентов, имеющих аллель 3669G (rs6198), отмечены более низкие уровни употребления сахара и общей энергетической ценности пищи, а также более высокая чувствительность к инсулину и меньшая тревожность [57]. Эти данные получены среди детей и подростков от 10 до 17 лет, которые находились под наблюдением в течение 5 лет. Полученные результаты подчеркивают, что эта генетическая вариация в гене ГР связана со снижением чувствительности к ГК, что на когнитивном и поведенческом уровнях приводит к изменению характера потребления пищи и реакций на эмоциональный стресс. Этот генетический вариант может сыграть важную роль в снижении риска метаболических и психиатрических заболеваний.

Описанные полиморфные варианты гена NR3C1 не единственные — есть и другие изменения гена ГР, которые сопровождаются относительной резистентностью или гиперчувствительностью к ГК и ассоциированы с изменением риска ССЗ, включая TthIII, D401H, A714Q, F737L, F774S, V575G, D641V и G679S [53]. Тем не менее их значение и фенотипы продолжают изучаться.

## ЗАКЛЮЧЕНИЕ

ГК часто назначаются при различных состояниях, однако их терапевтическая эффективность ограничивается нежелательными побочными явлениями. При этом часть эффектов ГК реализуется через ядерный рецептор. Насколько известно на настоящий момент, полиморфные варианты гена ГР NR3C1 ассоциированы с интенсивностью проявлений гиперкортицизма, обуславливая наблюдаемое разнообразие чувствительности к эндо- и экзогенным ГК, вариабельность метаболических нарушений. Синдром гиперкортицизма является констелляцией, увеличивающей вероятность фатальных сердечно-сосудистых событий. В связи с чем изучение вариаций гена ГР может помочь в раннем выявлении кардиометаболических нарушений, ССЗ и их прогнозировании.

## ДОПОЛНИТЕЛЬНАЯ ИНФОРМАЦИЯ

Источники финансирования. Работа выполнена по инициативе авторов без привлечения финансирования.

Конфликт интересов. Авторы декларируют отсутствие явных и потенциальных конфликтов интересов, связанных с содержанием настоящей статьи.

Участие авторов. Бровкина С.С. — существенный вклад в концепцию исследования, получение и анализ данных, интерпретация результатов, написание статьи; Джериева И.С. — существенный вклад в концепцию исследования, внесение в рукопись существенной правки с целью повышения научной ценности статьи; Волкова Н.И. — существенный вклад в дизайн исследования, внесение в рукопись существенной правки с целью повышения научной ценности статьи; Шкурат Т.П. — существенный вклад в концепцию исследования, внесение в рукопись существенной правки с целью повышения научной ценности статьи; Гончарова З.А. — существенный вклад в дизайн исследования, внесение в рукопись существенной правки с целью повышения научной ценности статьи; Машкина Е.В. — существенный вклад в дизайн исследования, внесение в рукопись существенной правки с целью повышения научной ценности статьи; Решетников И.Б. — интерпретация результатов, написание статьи. Все авторы одобрили финальную версию статьи перед публикацией, выразили согласие нести ответственность за все аспекты работы, подразумевающую надлежащее изучение и решение вопросов, связанных с точностью или добросовестностью любой части работы.
